# Addenbrooke's Hospital

**Published:** 1898-12-03

**Authors:** 


// The Hospital

A Journal of

The Medical Sciences and Hospital Administration.

Vol. XXV.—No. 636.] Edited by Sir Henry Burdett, K.C.B., and Solomon C. Smith, M.D., M.R.C.P. [Dec 3, 1898.

### Addenbrooke's Hospital.

The public mind, not only of Cambridge, but of Cambridgeshire, has for some time past been greatly exercised with regard to the future management of Addenbrooke's Hospital, in a manner which was happily set at rest on Monday by a poll of the governors, which gave a majority of fifty-one votes in favour of a proposal which originated with Sir Henry Burdett, and which, although it was denounced by Dr. Latham and others as "revolutionary," will now be put into practice and carried to its legitimate conclusions. The hospital had for some time past been in a more or less declining financial position, and Sir Henry was called in to advise as to the methods by which a better state of things might be brought about. Up to the present time the affairs of the hospital had been managed by a weekly meeting of the governors, a meeting which any governor might attend who pleased, and to which there was no special inducement to bring any who might prefer to stay away. This system is in force at only one of the London hospitals, where the governors are far more numerous, being in larger proportion resident within easy reach of the institution, and affording a comparatively greater number of men of leisure. At Cambridge the governors may be divided almost equally between the town, the University, and the county. Although the system has continued unchanged for very many years, its results have not given complete satisfaction, nor have they contributed to the financial well-being of the hospital. There was also the further danger that the decision of one meeting could be reversed at the next by anyone who procured the attendance of another set of governors. The natural effect has been to induce something which might perhaps be described as carelessness in respect of the innumerable details upon which the prosperity of an institution, be it a hospital or any other, must ultimately depend; with the natural result of a falling subscription list and of a diminution of that complete efficiency at which a hospital, now affording the materials of instruction to the pupils of a great and increasing medical school, should not only aim, but should be determined to secure.

The authorities, fully conscious that there was something wrong in their organisation or methods of working, called in the assistance of Sir Henry Burdett as a skilled adviser, and he presented a report, in which very sweeping changes in the constitution and mode of government were advocated. He advised that the loosely constituted Weekly Board, with the uncertain attendance of governors, should be replaced by a regularly appointed committee of eighteen members, with whom should rest not only the entire internal control of the establishment, but also a power to provide for the prompt payment of all demands upon the charity, or for the complete settlement of all accounts within the limits of each financial year. His suggestions, with one exception, were unanimously accepted by the special committee charged with the duty of reporting upon them to the governors; but were earnestly opposed by certain extremely well-meaning gentlemen, to whom, as they called the proposals of Sir Henry "revolutionary," the term "reactionary" may perhaps without offence be applied. The recommendations were defeated last week in a general meeting by a single vote in thirty-one, and a poll was at once demanded by the losing side, which, as we have said, has resulted in the acceptance of the first of Sir Henry Burdett's proposals, and so endorsed the principle underlying the whole of his recommendations. One of the most important of them is that the medical staff shall furnish a definite and sufficient contingent to the membership of the new committee, so that every proposal for the better government of the institution may have to run the ordeal of medical and surgical criticism before it can be adopted.

The one exception to the acceptance of the recommendations by the special committee is that Sir Henry advised the appointment of a resident superintendent, at a sufficient salary, whose business it would be to focus the details of administration, and to see that the accounts of the hospital were kept upon what is known as the "uniform" system, so as to admit of ready comparison with those of other institutions, and of the ready detection of the various leakages, through some or all of which it is possible to permit the funds of charities to be diverted from their proper uses, or to be expended without securing a due return. The appointment of such an officer will be voted upon at the adjourned meeting of the governors on the 5th inst. The figures furnished in connection with the controversy seem to show that there is abundant disposition on the part of residents in the University, the town and the county, to maintain their widely renowned hospital in complete efficiency, and, now that the new arrangements will afford increased certainty of careful, uniform, and economical management, there can be little doubt that this disposition will be increased. It is satisfactory to know that, with one exception, the members of the medical and surgical staff were not only unanimous, but active, in their support of Sir Henry Burdett's proposals, and we think there can be no question that they will be in a position before long to adduce conclusive evidence that their wisdom has been justified by its results.

